# Health Literacy and Active Transport in Austria: Results from a Rural Setting

**DOI:** 10.3390/ijerph17041404

**Published:** 2020-02-21

**Authors:** Kathrin Hofer-Fischanger, Bianca Fuchs-Neuhold, Alexander Müller, Gerlinde Grasser, Mireille N.M. van Poppel

**Affiliations:** 1 Institute of Health and Tourism Management, FH JOANNEUM University of Applied Sciences, Bad Gleichenberg 8344, Austria; bianca.fuchsneuhold@fh-joanneum.at (B.F.-N.); gerlinde.grasser@fh-joanneum.at (G.G.); 2 Institute of Sports Science, University of Graz, Graz 8010, Austria; alexander.mueller@medunigraz.at (A.M.);; 3 Division of Endocrinology and Diabetology Department of Internal Medicine, Medical University of Graz, Graz 8036, Austria

**Keywords:** health literacy, active transport, active mobility, rural area

## Abstract

Health literacy (HL) has been determined for the general population and for subgroups, though the relationship between HL and active transport in rural areas was not explored. The aim of our study is to investigate HL among citizens in an Austrian rural region and to explore the associations between HL and active transport. This cross-sectional telephone survey included 288 adults (171 women) with a mean age of 57.8 (SD 0.9). HL was assessed using the HLS-EU-Q16 questionnaire. Active transport was measured as the minutes per week spent on walking or cycling from A to B. After descriptive analysis, the association between HL and active transport was assessed using linear regression models. The mean HL score for all participants was 37.1 (SD 7.7). Among all subjects, 6.9% showed inadequate HL, 25.7% problematic HL, 38.9% sufficient HL, and 28.5% excellent HL. HL was significantly higher among citizens with high education (*p* = 0.04) and training/employment in healthcare (*p* = 0.001). Active transport was not associated with HL (*p* = 0.281). Active transport in rural areas might be influenced by other predictors like distance to work, street connectivity, and accessible facilities for walking and biking. This needs to be explored further for rural areas.

## 1. Introduction

Improving health literacy (HL) is a common public health approach and deals with the empowerment of individuals, organizations, and communities for more independent decision-making in health topics [1]. Sørensen et al. [2] defined HL as a comprehensive concept which is “people’s knowledge, motivation, and competences to access, understand, appraise, and apply health information”. An excellent HL enhances the quality of life due to active judgments and decisions concerning healthcare, disease prevention, and health promotion in everyday life [1,2,3].

Results for HL for the general population were published first in 2015 in eight European member states [4]. In this study, huge differences in HL between countries were shown. The proportion of respondents with inadequate or problematic HL was lowest in the Netherlands (29%) and highest for Bulgaria (62%). In Austria, 56.4% of respondents reported inadequate or problematic HL [5,6].

More recently, HL was measured for specific population groups in Germany [7,8], the Netherlands [9], or the European Union (EU) [10], e.g., children and adolescents, elderly people, or migrants. In addition, the influence of various factors, such as health behavior (physical activity, smoking, and eating habits) or health-related factors (body mass index (BMI), blood pressure, chronic diseases, and pain), of HL was assessed [5,7,8,11,12]. Previous studies showed a positive relationship between HL and physical activity [9,11,13,14]. The participation rate in moderate to vigorous physical activity as well as frequency and duration of physical activity during HL oriented type 2 diabetes (T2D) programs increased among patients with high HL [15,16]. On the other hand, low HL was associated with physical limitations, e.g., difficulty in performing everyday activities [17,18]. A recent study reported a positive correlation between some of the HL subscales and daily physical activity, but not of overall HL [19].

Little is known about the relationship between HL and active transport, which is physical activity for everyday transport by walking or cycling. Active transport is a strong determinant for health and important in health promotion. An increase in walking and cycling for transportation is related to positive health effects, e.g., better mental health, a lower body weight, a reduction of chronic diseases, and a better general cardiovascular health status [20,21,22].

Previous studies reported urban–rural differences for active transport. Residents in rural areas take fewer steps per day and spend fewer minutes per week walking (walking for leisure or transportation) compared to urban residents. Residents in rural areas also cycled less for transport [23,24]. As the body mass index (BMI) of a citizen in high-income countries (central and eastern Europe) is higher in rural than in urban areas, interventions promoting physical activity and active transport can contribute highly to general population health [25]. Besides health benefits, active transport can make a contribution to reducing greenhouse gas emissions and can bring economic benefits by a reduction of healthcare costs [26,27]. Although people with better HL are more physically active than those with low HL [13], currently it is unknown if they are also more likely to choose active transport over passive.

Thus, we designed the present study to examine HL among citizens in an Austrian rural region and to determine the associations between HL and active transport. This aim fits well with the Austrian Health Targets that focus on enhancing HL (target 3) as well as promoting physical activity in everyday life (target 8) [28].

## 2. Materials and Methods

### 2.1. Study Design

This cross-sectional study was conducted in July 2018 in a rural area with 13,100 inhabitants in southeast Austria. Rurality is defined by OECD [29] as an area of communities with a low population density (<150 inhabitants per km^2^) and which does not include an urban center. This definition applies to the rural area of the present study.

From 6300 households in the region, 300 were randomly selected from the telephone directory. For the first 200, the person who answered the telephone was interviewed, when aged >18 years. To make the study sample as representative as possible, in the last 100 households, the youngest eligible person (>18 years) in the household was asked to complete the interview. People who did not appear in the telephone directory, did not live in the region, or were aged under 18 years were excluded.

The sample size of 300 was based on feasibility. Assuming that associations between HL and active transport would be weak (effect size of 0.2), and with a two-sided alpha of 5%, a minimum of 55 participants was needed to achieve 90% statistical power.

The survey was developed on the basis of two validated health-related questionnaires (SF-12, HLS-EU-Q16) [4,30] and further questions on active transport and health taken from scientific literature [21,23]. Our questionnaire consisted of questions regarding HL and questions on active transport in everyday life, sociodemographic and anthropometric variables like weight and height, as well as self-perceived health status.

The study was approved by the local ethics committee of the University of Graz (GZ. 39/70/63 ex 2017/18).

### 2.2. Health Literacy (HL) Measures

HLS-EU-Q16 is the short version of HLS-EU-Q47 and measures health literacy (HL) based on the definition from Sørensen with three domains: healthcare, disease prevention, and health promotion [5]. It is a self-reported tool with responses in Likert-type (“very easy”, “fairly easy”, “fairly difficult”, “very difficult”). HL index score was computed based on the validated HLS-EU-Q16 questionnaire [4]. The index score was calculated for those respondents who answered ≥80% (i.e., ≥13 items) of the questionnaire. For comparison with other studies, the following formula was used to calculate the HL score [31]:

Index=(mean (per Item)−1)∗50/3

The index score has a minimum value of 0 and a maximum value of 50. In addition, four categories were formed on the basis of the score as defined by Sørensen et al. [6] (0–25 inadequate HL; >25–33 problematic HL; >33–42 sufficient HL, and >42–50 excellent HL).

### 2.3. Socio-Demographic and Health-Related Variables

Participants were asked about educational level, net household income, migration background, employment status, marital status, and whether they were trained or employed in healthcare.

The educational level was categorized according to the International Standard Classification of Education [32] as follows: low (primary school or lower secondary school without vocational training), medium (vocational training, upper secondary school, or professional school), high (any higher level, e.g., tertiary education). The net household income of participants (per month) was classified into three categories: <€1850 (low income), €1850 to €2950 (medium income), and >€2950 (high income). Participants were considered to have a migration background if they or one of their parents were not born in Austria. Employment status was classified into three categories: employed (fully employed or part-time), retired, or other (special-order contract, unemployed, maternity leave, student, or without income). Marital status was classified as married/living together, or single. Participants were considered to have training or employment in healthcare when they had an educational background or actual/former employment in the field of healthcare.

Self-reported height and weight of participants were assessed in order to calculate their body mass index (BMI) as weight in kg divided by height in meters squared. BMI was categorized into standard BMI categories: underweight (<18.5 kg/m^2^), normal weight (18.5–24.9 kg/m^2^), overweight (25.0–29.9 kg/m^2^), and obese (≥30.0 kg/m^2^). Furthermore, the self-perceived health status was measured with SF-12 [30].

### 2.4. Active Transport

Based on previous work [21,23], participants were asked “Which of the following transport options did you choose for commuting in the last 12 months?” followed by seven subcategories: (1) foot; (2) car; (3) public transport; (4) bicycle or freight bicycle; (5) electric bicycle; (6) motorbike or moped; (7) taxi. Subcategories were answered with the following response options regarding frequency of active transport: daily, 3–4 days per week (daily/often), 1–2 days per week, 1–3 days per month (weekly/1–3 times a month), seldom, never (seldom/never), do not know.

In addition, we measured active transport as the minutes per week spent on walking or cycling from A to B. For walking, participants were asked “On how many days in a usual week do you walk more than 10 minutes from A to B?” and “For how long do you walk on such a day?”. Participants reported a number of days per week walking. For cycling (including electric bicycling) the same set of questions was asked.

Total minutes of walking per week was calculated by multiplying the number of hours reported with sixty and adding them to the minutes reported. Consequently, minutes of active walking per week was calculated by multiplying the reported number of days per week with the calculated minutes per day. This number of minutes of walking for active transport per week was used in the analyses. Minutes of cycling for active transport per week were calculated with the same steps.

### 2.5. Statistical Analysis

Continuous variables with a normal distribution are displayed as means with their standard deviation (SD). When distribution was not normal, median and interquartile range (IQR) are reported. Categorical variables are displayed as numbers (n) and percentages (%). Significance of differences between subgroups (based on gender, age, educational level, healthcare training/employment, active transport etc.) in HL score [31] was tested with independent t-test since HL was normally distributed. The association between active transport and HL was calculated with linear regression models, using the HL score as an independent variable and active transport as the dependent variable. Models were adjusted for variables that might influence the association between HL and active transport, and were chosen based on our own data (education, healthcare training/employment) and on literature (gender and age). Unadjusted models and adjusted models, including confounding variables, are presented. In the last step, the variable healthcare training/employment was added as another possible confounding variable. The regression coefficients resulting from linear regression are displayed with their 95% confidence interval (95% CI).

A *p*-value of <0.05 was considered statistically significant. All analyses were performed using IBM SPSS Statistics for Windows, Version 24.

## 3. Results

In total, 288 adults (171 women; 59.4%) could be included in the analysis. Twelve (4%) participants were excluded because they answered <80% of the items of the HLS-EU-Q16. The mean age of the subjects was 57.8 (SD 0.9) years. Demographic, socio-economic, anthropometric, and health-related characteristics are shown in Table 1.

Three-quarters of the study population had a medium level of education and 35% of women had a monthly household income under €1850. Only 4.5% had a migration background. Most participants were either employed (42%) or retired (47.2%). About two-thirds were married or living together with a partner and 18% showed a connection to healthcare because of training or former employment in this field. The mean BMI of the subjects was 25.9 (SD 4.1). In total, 41.7% of all participants were overweight (33.3% of women, 57.0% of men) and 13.6% were obese. The majority of participants rated their health status as very good (30.2%), or good (43.8%).

### 3.1. Active Transport Characteristics

Overall, participants reported a median of 180.0 (IQR 90; 410) minutes per week (min/wk) of active transport (walking 162.5 (IQR 60; 300) min/wk; cycling 105.0 (IQR 45; 240) min/wk; see Table 2). Men reported more active transport in general, for walking, cycling and electric cycling than women, however, this difference was not statistically significant. Participants walked on 4.3 (SD 2.3) days per week and cycled on 3.4 (SD 2.1) days per week on average. Seventy percent of men walked daily or often, while only 50% of the women did so. There were no differences between men and women for the frequency of cycling.

### 3.2. Health Literacy (HL) Score, Socio-Demographics, and Active Transport

The mean HL score of 288 study participants was 37.1 (SD 7.66), and 6.9% had inadequate HL, 25.7% problematic HL, 38.9% sufficient HL, and 28.5% had excellent HL (Table 3).

The HL score was not significantly different between men and women, between age groups, or between employed and retired participants. The HL score was related to education. Participants with high education had a 3.6 points higher score (95% CI 0.18; 7.11, *p* = 0.04) compared to participants with low education.

HL was related to training or employment in healthcare. Participants with training or employment in the health sector had a 3.8 points higher HL score (95% CI 1.51; 6.03, *p* = 0.001) compared to participants with no health training or employment.

Regarding the frequency of active transport by walking (*p* = 0.94) or cycling (*p* = 0.11), no significant differences between the groups were found. Respondents with sufficient HL had 34.5 min/wk more active transport compared to participants with problematic HL, but this was not statistically significant.

### 3.3. Health Literacy (HL) and Active Transport

The results of linear regression models for the association between HL and active transport are shown in Table 4. We did not observe an association between HL and active transport in terms of weekly minutes of walking and cycling. Adjustment for possible confounding variables did not change the results. The variables of gender, age, educational level, and training/employment in healthcare were not significantly associated with active transport. Also, the association between HL and active transport was not modified by age, gender, or education (*p*-value for interaction terms all >0.05).

## 4. Discussion

We aimed to describe HL among citizens in an Austrian rural region and to explore associations between HL and active transport. Standardized measurement tools were used, for HL from the HLS-EU project and for active transport from previous studies in this field. No association between HL and active mobility overall, nor by walking or cycling separately, was found.

### 4.1. Active Transport and HL

The study participants reported on average 180.0 min/wk (162.5 walking and 105.0 cycling) for active transport. Men had, in general, more active transport than women, also for walking and cycling separately, although this difference was not statistically significant. Respondents walked on 4.3 days/wk and cycled on 3.4 days/wk for more than 10 min from A to B. This reported level of active transport is surprisingly high. In comparison to an Austrian study conducted in the city of Graz (median 150 min/wk) our respondents reported more active transport [33]. In that study, men also had more active transport compared to women. The IPEN study also showed lower rates of active transport in Denmark (walking 3.3 days/wk; cycling 2.4 days/wk) and Belgium (walking 2.0 days/wk; cycling 1.7 days/wk) [34]. However, comparisons between countries are complex, because of differences between infrastructural and cultural factors.

Former studies reported a lower frequency of active transport in rural neighborhoods compared to urban [21,23,35], but with a higher daily duration [21]. A rural area may provide fewer opportunities for short-distance trips. This might lead to less active transport, since people are less likely to choose an active mode of transport if the destination is not within 800 m of walking distance [36,37].

We could not show that active transport in terms of weekly minutes of walking and cycling is influenced by HL. The result did not change after adjustment for confounding variables (gender, age, educational level, and training/employment in healthcare). Previous studies described street connectivity, bike lane connectivity, land-use mix, or walkable neighborhoods as predictors for walking and cycling, although they focused on urban areas [34,38,39]. Moreover, the configuration of the natural environment (ground conditions, hills) and aspects of traffic safety (high density and speed of traffic) also influence levels of active transport [36,37,40].

In rural areas, the built environment might be a barrier for active transport, due to a lack of connection between streets, long distances, and low walkability and bike-ability (caused by the absence of footpaths, cycle lanes, or streetlights at night). These barriers might be more important and preclude finding an association between HL and active transport in a rural area.

In our study population, we found an increase in HL in higher educated participants and a non-significantly higher HL in respondents who cycle “weekly/1–3 times a month” compared to those who cycle “daily/often”. The association between education and active transport shown in the literature is controversial [38,41,42]. In a rural context as in our study, many higher educated citizens might not find work in their living area, and thus, have a longer distance to work and need to commute by car. Therefore, they might not be able to walk or cycle much on working days, but can have active transport during the weekend. Unfortunately, we did not have information on commuting distance. The possible interaction between education and HL in the association with active transport, especially in rural areas, needs to be explored in further research.

### 4.2. HL and Baseline Characteristics

We found higher HL in our study population compared to former studies on EU and country-level using the same questionnaire (HLS-EU 16). The mean HL score in the HLS-EU study was 32.0 for Austria and 33.8 over eight countries [6], compared to 37.1 in our study population. However, the mean age in our study population was 57.8 years which is much higher than in the HLS-EU project. Previous studies in Germany and country-specific results of the HLS-EU survey (e.g., Netherlands) show an increase of HL score with age [8,43]. The rural setting might also be an explanation for the higher HL score in our study. The questionnaire measures, based on the definition from Sørensen, the ability of patients to navigate or negotiate the health care system [1]. Possibly, it could be rather easy for citizens in the rural region of our study to orient themselves in the system and navigate between the small number of health care providers (e.g., one hospital, a few doctors or only some other healthcare professionals). Therefore, the opportunities for healthcare choices or treatments are clearer and possibly more manageable. This assumption needs further investigation.

In our study population, no difference in HL score was found between men and women. Previous studies found controversial results regarding the association of HL and gender. A meta-analysis from 2005 concluded that there are no gender differences in HL [44]. However, Pelikan et al. found a slightly higher HL for women in the EU study population as well as in the Austrian sample [43].

We found an association between HL and education as well as with training/employment in healthcare. Participants with low education had a significantly lower HL score than subjects with high education level. In addition, the HL score of participants with an educational background or an actual/former employment in the field of healthcare was significantly higher. A strong connection for HL and educational level was also found in the HLS-EU survey [43] and several former studies [6,8,11,12,17,18,44]. Lorini et al. [12] also showed in their Italian HL survey that being trained or employed in the field of healthcare is associated with a higher HL score.

### 4.3. Limitations of the Study

To our knowledge, this is the first study analyzing HL in a rural area as well as exploring associations between HL and active mobility.

Due to the fact that it was a telephone survey, some limitations need to be recognized. Firstly, people who did not appear in the telephone directory could not be part of the study. This pertains especially to younger people who are often not registered with their mobile number. However, the study sample was representative of the Austrian population with regards to gender, education, and BMI. The comparison of study results with former studies could be influenced by the interviewing method. Former studies like HLS-EU used the method of computer-assisted personal interviewing (CAPI) [5]. For reasons of time, we used computer-assisted telephone interviewing (CATI). The questionnaire for HL consisted of 16 subquestions, all with the same Likert scale answer options. This set of questions was asked at the end of the interview, which could have influenced attention and motivation for answering each question accurately. Therefore, the instrument for measuring HL might need to be validated specifically as a telephone survey. The data for HL and active transport was self-reported and did not include any objective items to measure functional HL, which might impact its accuracy. Previous studies reported differences between self-reported and accelerometer-measured data on active mobility [45]. Another limitation of our study was the unavailability of data for distances for commuting.

It would have been interesting to compare our findings with other rural data. As it was the first study to explore HL in a rural setting this was not possible. Although previous studies analyzed active transport in rural areas, the comparison with studies from different countries is difficult due to a different understanding of the term “rural”.

## 5. Conclusions

This study shows a higher HL score for the rural study population compared to national data from the HLS-EU survey. HL was associated with education and being trained or employed in the field of healthcare. Previous studies showed a relationship between HL (or sub-dimensions of HL) and total physical activity. It could not be shown in our study that citizens with high HL are also more likely to choose active over passive transport. Active transport in rural areas might be influenced by other predictors like distance to work, street connectivity, and accessible facilities for walking and biking. This needs to be explored further for rural areas.

## Figures and Tables

**Table 1:** Baseline characteristics of the study population.

	N = 288		Female		Male	
	Proportion in % Mean (SD)	n	Proportion in % Mean (SD)	n	Proportion in % Mean (SD)	n
Age (yrs ^1^)	57.8 (0.9)	288	57.9 (1.1)	171	57.7 (1.4)	117
<55 yrs	41.0	118	40.4	69	41.9	49
≥55 yrs	59.0	170	59.6	102	58.1	68
Educational level						
Low education	9.4	27	12.3	21	5.1	6
Medium education	76.0	219	74.3	127	78.6	92
High education	14.6	42	13.5	23	16.2	19
Household income (n = 228)						
<1850 EUR	35.1	80	40.5	53	27.8	27
1850 EUR–2950 EUR	32.0	73	30.6	40	34.0	33
>2950 EUR	32.9	75	29.0	38	38.2	37
Migration background						
yes	4.5	13	6.4	11	1.7	2
Emloyment status						
Employed	42.0	121	38.6	66	47.0	55
Retired	47.2	136	47.4	81	47.0	55
Other (e.g. student, unemployed)	10.7	31	14.0	24	6.0	7
Marital status						
Married or living together	70.5	203	73.1	125	66.7	78
Trained or employed in healthcare						
yes	18.4	53	23.4	40	11.1	13
BMI ^2^ (kg/m^2^)	25.9 (4.1)	279	25.5 (4.6)	165	26.7 (3.1)	114
Underweight	0.7	2	1.2	2	0	0
Normal weight	41.0	118	52.1	86	28.1	32
Overweight	41.7	120	33.3	55	57.0	65
Obese	13.6	39	13.3	22	14.9	17
Self-perceived health status						
very good	30.2	87	31.0	53	29.1	34
good	43.8	126	44.4	76	42.7	50
fair	23.3	67	22.2	38	24.8	29
poor	2.8	8	2.3	4	3.4	4

^1^ years; ^2^ Body Mass Index.

**Table 2:** Active transport of the study population.

	Total Sample	n	Female	n	Male	n
Active transport min/wk ^1^, median (IQR ^2^)	180.0 (90; 410)	233	180.0 (90; 379)	134	210.0 (100; 420)	99
Active walking min/wk	162.5 (60; 300)	214	140.0 (60; 278)	120	180.0 (76; 300)	94
Active cycling min/wk	105.0 (45; 240)	116	105.0 (45; 225)	67	120.0 (35; 240)	49
Active electric cycling min/wk	120.0 (48; 285)	25	120.0 (45; 420)	17	135.0 (70; 203)	8
Frequency of active transport/wk, mean (SD)						
frequency of walking days/wk ^3^	4.3 (2.3)	214	4.1 (2.4)	120	4.6 (2.2)	94
frequency of cycling days/wk	3.4 (2.1)	116	3.4 (2.1)	67	3.4 (2.1)	49
General frequency of walking %						
daily/often	58.0	167	49.7	85	70.1	82
weekly/1–3 times a month	20.8	60	25.7	44	13.7	16
seldom/never	21.2	61	24.6	42	16.2	19
General frequency of cycling, %						
daily/often	27.4	79	26.9	46	28.2	33
weekly/1–3 times a month	22.9	66	24.6	42	20.5	24
seldom/never	49.3	142	48.5	83	50.4	59

^1^ minutes per week; ^2^ interquartile range, ^3^ days per week.

**Table 3:** Distribution of health literacy (HL) levels and HL scores of the study population.

	Number (%)				
Categories	Inadequate (0–25)	Problematic (>25–33)	Sufficient (>33–42)	Excellent (>42)	Mean (SD) HL Score
Total	20 (6.9%)	74 (25.7%)	112 (38.9%)	82 (28.5%)	37.1 (7.66)
Sex					
Female	10 (5.8%)	44 (25.7%)	64 (37.4%)	53 (31.0%)	37.5 (7.62)
Male	10 (8.5%)	30 (25.6%)	48 (41.0%)	29 (24.8%)	36.6 (7.74)
Age					
<55 years	7 (5.9%)	30 (25.4%)	45 (38.1%)	36 (30.5%)	37.4 (7.94)
≥55 years	13 (7.6%)	44 (25.9%)	67 (39.4%)	46 (27.1%)	37.0 (7.49)
Educational Level					
Low	2 (7.4%)	7 (25.9%)	11 (40.7%)	7 (25.9%)	36.5 (7.25)
Medium	17 (7.8%)	64 (27.9%)	85 (38.8%)	56 (25.6%)	36.6 (7.75)
High	1 (2.4%)	6 (14.3%)	16 (38.1%)	19 (45.2%)	40.2 (6.90) *
Migration background					
Yes	1 (7.7%)	1 (7.7%)	6 (46.2%)	5 (38.5%)	39.3 (8.06)
No	19 (6.9%)	73 (26.5%)	106 (38.5%)	77 (28.0%)	37.0 (7.64)
Employment status					
Employed	8 (6.6%)	36 (29.8%)	39 (32.2%)	38 (31.4%)	37.3 (8.13)
Retired	10 (7.4%)	34 (25.0%)	54 (39.7%)	38 (27.9%)	37.1 (7.60)
Trained/employed in hc ^1^					
yes	3 (5.7%)	10 (18.9%)	13 (34.5%)	27 (50.9%)	40.2 (8.12) *
no	17 (7.2%)	64 (27.2%)	99 (42.1%)	55 (23.4%)	36.4 (7.41)
Frequency of walking					
daily/often	13 (7.8%)	40 (24.0%)	71 (42.5%)	43 (25.7%)	37.0 (7.75)
weekly/1–3 times a month	3 (5.0%)	18 (30.0%)	20 (33.3%)	19 (31.7%)	37.7 (7.56)
seldom/never	4 (6.6%)	16 (26.2%)	21 (34.4%)	20 (32.8%)	37.0 (7.63)
Frequency of cycling					
daily/often	7 (8.9%)	17 (21.5%)	36 (45.6%)	19 (24.1%)	37.1 (7.44)
weekly/1–3 times a month	1 (1.5%)	14 (21.2%)	32 (48.5%)	19 (28.8%)	38.5 (6.73)
seldom/never	11 (7.7%)	43 (30.3%)	44 (31.0%)	44 (31.0%)	36.6 (8.12)
Active transport					
mean min/wk ^2^ (SD)	210.3 (196.2)	205.2 (189.8)	239.7 (188.8)	221.8 (177.8)	-

^1^ healthcare; ^2^ minutes per week; * *p* < 0.05.

**Table 4:** Associations for total active transport as the dependent variable and HL as the independent variable, adjusted for gender, age, educational level, and training/employment in healthcare.

	Total Sample		
	ß	95% CI	*p*
**Model 1**			
HL Score	0.012	–0.010; 0.034	0.281
**Model 2**			
HL Score	0.013	–0.009; 0.035	0.257
Gender			
female	*ref.*		
male	–0.163	–0.512; 0.186	0.359
Age	0.006	–0.006; 0.017	0.314
Educational Level			
low	*ref.*		
medium	0.070	–0.537; 0.677	0.820
high	0.101	–0.619; 0.821	0.783
**Model 3**			
HL Score	0.014	–0.008; 0.037	0.208
Gender			
female	*ref.*		
male	–0.133	–0.486; 0.221	0.460
Age	0.006	–0.006; 0.017	0.324
Educational Level			
low	*ref.*		
medium	0.087	–0.521; 0.694	0.779
high	0.167	–0.563; 0.897	0.653
Trained / Employed in hc ^1^			
yes	*ref.*		
no	0.251	–0.717; 0.216	0.291

^1^ healthcare.
